# L1CAM Is a Marker for Enriching Corticospinal Motor Neurons in the Developing Brain

**DOI:** 10.3389/fncel.2020.00031

**Published:** 2020-02-19

**Authors:** Bumpei Samata, Rika Takaichi, Yuko Ishii, Kaori Fukushima, Harumi Nakagawa, Yuichi Ono, Jun Takahashi

**Affiliations:** ^1^Department of Clinical Application, Center for iPS Cell Research and Application, Kyoto University, Kyoto, Japan; ^2^Department of Developmental Neurobiology, KAN Research Institute Inc., Kobe, Japan

**Keywords:** L1 cell adhesion molecule, corticospinal motor neurons, transplantation, corticospinal tract, cell sorting

## Abstract

The cerebral cortical tissue of murine embryo and pluripotent stem cell-derived neurons can survive in the adult brain and extend axons to the spinal cord. These features suggest that cell transplantation can be a strategy to reconstruct the corticospinal tract (CST). It is unknown, however, which cell population makes for safe and effective donor cells. To address this issue, we grafted the cerebral cortex of E14.5 mouse to the brain of adult mice and found that the cells in the graft extending axons along the CST expressed CTIP2. By using CTIP2:GFP knock-in mouse embryonic stem cells (mESCs), we identified L1CAM as a cell surface marker to enrich CTIP2^+^ cells. We sorted L1CAM^+^ cells from E14.5 mouse brain and confirmed that they extended a larger number of axons along the CST compared to L1CAM^−^ cells. Our results suggest that sorting L1CAM^+^ cells from the embryonic cerebral cortex enriches subcortical projection neurons to reconstruct the CST.

## Introduction

Reconstruction of the corticospinal tract (CST) by cell transplantation is one of the main strategies to treat brain injury and stroke. Previous studies have reported that behavioral improvement was observed after transplantation of the embryonic cerebral cortex in rat models with brain injury or middle cerebral artery occlusion (Plumet et al., 1993; Grabowski et al., 1995; Mattsson et al., 1997; Riolobos et al., 2001). Furthermore, retrograde labeling of the CST revealed that the grafted cortical tissue contributed to the reconstruction of the adult brain (Gaillard et al., 2007). Recently, cerebral neurons have been induced from both mouse embryonic stem cells (mESCs) and human-induced pluripotent stem cells (hiPSCs) by recapitulating corticogenesis (Gaspard et al., 2008; Espuny-Camacho et al., 2013). When grafted into the frontal cortex, the induced neurons extend their axons to the corresponding targets and integrate into the host brain (Espuny-Camacho et al., 2013). Thus, reconstruction of the CST by PSC-derived neurons can be a novel therapy for brain injury and stroke.

The developing cerebral cortex is a complex structure consisting of various neurons and neural progenitors. When fetal cortical tissue is grafted into the frontal cortex, most surviving neurons extend axons to other cortical areas of the brain (callosal projections), with only a small population extending axons beyond the cortex (subcerebral projections; Ballout et al., 2016). Furthermore, there is a risk of tumor formation by the progenitor cells, especially in the case of PSCs. Thus, for successful cell-based therapy, it is important to enrich subcerebral projection neurons, especially corticospinal motor neurons (CSMNs), as the donor cell population.

The sorting of dopaminergic progenitors has been shown to be useful for cell-based Parkinson’s disease therapies in terms of safety and efficiency (Doi et al., 2014; Samata et al., 2016). However, there are no reports about a similar method for the sorting of CSMN progenitor cells. Here, we identified a cell surface marker for such progenitor cells and show sorting with this marker enhances the survival of donor cells in the brain and extension of axons along the CST after transplantation.

## Materials and Methods

### Animals

All animal experiments were performed according to the guidelines for Animal Experiment of Kyoto University, the guidelines for the Care and Use of Laboratory Animals of the Institute of Laboratory Animal Resources (ILAR; Washington, DC, USA) and the guideline for the Animals in Research: Reporting *in vivo* Experiments (ARRIVE). Sixteen week-*old* female nude rats (*F344/NJcl-rnu/rnu*) were obtained from CLEA Japan, Inc. (Tokyo, Japan). Nine week-*old* male mice (*C57BL/6NCrSlc*) and pregnant female mice (*C57BL/6NCrSlc* and *C57BL/6* *6-Tg*) were obtained from Shimizu Laboratory Supplies Company Limited (Kyoto, Japan). All animals were housed under diurnal lighting conditions (12 h light/12 h dark) and given standard food and water *ad libitum*.

### Transplantation Into Adult Animals

We anesthetized the animals with isoflurane (Intervet Inc., Tokyo, Japan), and the motor cortex was aspirated from 0.5 to 2.0 mm lateral to the midline and from 0.5 to 2.0 mm rostral to the Bregma on the corpus callosum of the left hemisphere of each donor’s brain. Seven days after the aspiration, we isolated E14.5 mouse frontal cortex from GFP Tg mice and enzymatically digested the cortices using Accumax (Innovative Cell Technologies, Inc., San Diego, CA, USA) for 10 min at 37°C. The cell density was adjusted to 10^5^ cells/μl in DMEM/F12 [Fujifilm Wako Pure Chemical Corporation (Fujifilm)., Osaka, Japan] supplemented with 2 mM L-glutamine (L-Gln; Merck Ltd., Tokyo, Japan), 0.1 mM 2-mercaptoethanol (2-ME; Fujifilm), 1% (vol/vol) N2 supplement (Thermo Fisher Scientific Inc., Tokyo, Japan), 2% (vol/vol) B-27 supplement (Thermo Fisher Scientific), 1% (vol/vol) Penicillin-Streptomycin (PS; Merck) and 10 μM Y-27632 (Fujifilm). The cell suspension (2 μl/site) was injected into the host brain. Before the sorted cell aggregates were injected into the brain, they were replated in a prime surface 96-well plate (Sumitomo Bakelite Company Limited, Tokyo, Japan) at a density of 3 × 10^4^ cells per well in 200 μl of DMEM/F12 containing 2 mM L-Gln, 0.1 mM 2-ME, 1% N2 supplement, 2% B-27 supplement, 1% PS and 10 μM Y-27632. Two days later, the cell aggregates were adjusted to 2 × 10^5^ cells (approximately 7–11 cell aggregates) and were placed into the host brain. After the transplantation, the animals were sacrificed and prepared for immunofluorescence studies.

### Retrograde Labeling

Fast blue (FB; Polysciences Inc., Warrington, PA, USA) was used for the retrograde labeling studies. To label cells that extend axons along the CST, FB solution containing 4% (vol/wt) FB, 4% (vol/vol) dimethyl sulfoxide (Merck) and artificial cerebrospinal fluid (Harvard Apparatus Inc., Holliston, MA, USA) was injected into the pyramidal decussation 7 days before sacrifice.

### Immunostaining

To stain the brain slices, animals transplanted with mouse cortex or sorted cells were sacrificed with pentobarbital (Tokyo Chemical Industry Company Limited, Tokyo, Japan) and perfused with 4% paraformaldehyde (PFA; Fujifilm) overnight, and then replaced with 10% (wt/vol) and 20% (wt/vol) sucrose (Nacalai Tesque Inc., Kyoto, Japan) in PBS overnight, respectively. The fixed brains were embedded in OCT compound (Sakura Finetec Japan Company Limited, Tokyo, Japan) and cut with a cryostat (CM-3050; Leica Inc.) at 16–30 μm thickness. The brain slices were placed into distilled water (DW; Thermo Fisher Scientific) containing 30% (vol/vol) ethylene glycol (Fujifilm), 30% (vol/vol) glycerol (Nacalai Tesque), 0.243% (wt/vol) NaH_2_PO_4_, 0.874% (wt/vol) Na_2_HPO_4_ and 0.34% (wt/vol) NaCl at −20°C until use. To stain the cell aggregates, the cell aggregates were perfused with 4% PFA for 15 min and then replaced with 10% (wt/vol) sucrose in PBS overnight. The fixed cell aggregates were embedded in OCT compound and cut with a cryostat at 16 μm thickness. The slices were attached on the surface of a MAS-coated slide glass (Matsunami Glass Inc Ltd., Osaka, Japan) and stocked at −20°C until use. To stain the cultured cells, the cells were perfused with 4% PFA for 15 min and washed with PBS. The fixed cells were stocked at 4°C until use.

The samples were permeabilized in PBS containing 2% (vol/vol) Triton X-100 (PBST; Nacalai Tesque) for 30 min. Then the samples were blocked with PBS containing 4% (wt/vol) Block Ace (Sumitomo Dainippon Pharma Company Limited, Tokyo, Japan) and 0.1% (vol/vol) Triton X-100 for 30 min. The primary antibodies were diluted in 0.1% (vol/vol) PBST containing 4% (wt/vol) Block Ace and incubated overnight at 4°C. The samples were washed with 0.1% (vol/vol) PBST and incubated with secondary antibodies in 0.1% (vol/vol) PBST containing 4% (wt/vol) Block Ace for 60 min at room temperature (RT), followed by washing with 0.1% PBST and incubating with 4′-6-diamidino-2-phenylindole (DAPI; Thermo Fisher Scientific) for 5 min at RT. Finally, the samples were mounted with DW containing 44% (vol/vol) 200 mM Tris-HCl (Nacalai Tesque), 22% (vol/vol) glycerol (Fujifilm), 0.02% (wt/vol) 1,4-diazabicyclo (2,2,2) octane (DABCO; Fujifilm) and 0.09% (wt/vol) Mowiol 4–88 reagent (Merck).

The primary antibodies used are as follows: anti-GFP (1:1,000; #598, Medical and Biological Laboratories Company Limited, Nagoya, Japan and 1:1,000; #04404-26, Nacalai Tesque), anti-CTIP2 (1:200; #12120S, Cell Signaling Technology Inc., Tokyo, Japan and 1:1,000; #ab18465, Abcam Inc., Cambridge, MA, USA), anti-FOXG1 (1:500; #ab18259, Abcam), anti-GSH2 (1:5,000; a kind gift from Dr. Mototsugu Eiraku, RIKEN, CDB), anti-M2 [1:100; #AB531785, Developmental Studies Hybridoma Bank (DSHB)], anti-M6 (1:100; AB2149607, DSHB), anti-L1CAM (1:1,000; #MAB5674, R&D Systems, Inc., Minneapolis, MN, USA; 1:500; #554273, BD Biosciences, San Jose, CA, USA), anti-NRP1 (1:500; #NP2111, ECM Biosciences, Versailles, KY, USA), anti-TBR2 (1:500; #ab23345, Abcam), anti-PAX6 (1:1,000; PRB-278P, BioLegend, San Diego, CA, USA; 1:500; #561462, BD Biosciences) and anti-CUX1 (1:200; #sc-13024, Santa Cruz Biotechnology, Santa Cruz, CA, USA). Alexa fluorescent-conjugated antibodies (1:500; Thermo Fisher Scientific) were used as secondary antibodies.

### Production of CTIP2:GFP Knockin (KI) mESC Line

A CTIP2:GFP KI targeting vector was assembled using pBS-IRES-GFP-polyA-neo-DTA. A 4-kb 5′-arm genomic fragment containing an ORF region of the exon 4 of the Ctip2 gene and a 3.5-kb 3′-arm fragment just downstream of the stop codon of Ctip2 were amplified by PCR using 129SV genomic DNA as a template and separately cloned into the NotI/XhoI and SalI/SpeI sites of pBS-IRES-GFP-polyA-neo-DTA to generate the targeting vector. CTIP2:GFP KI mESCs were generated by homologous recombination in the 129SVEV ESC line according to standard procedures and genotyped by PCR. Because the presence of the Neo cassette did not affect reporter gene expression in a similar Bcl11b-YFP KI mouse (Kueh et al., 2016), we used CTIP2:GFP KI ESCs containing the Neo cassette.

### Cell Culture

mESCs (EB5; passages 35–45) and CTIP2:GFP KI mESCs (passages 11–21) were maintained on a mitotically inactivated mouse embryonic fibroblast feeder layer in KnockOut DMEM (Thermo Fisher Scientific) supplemented with 20% (vol/vol) Fetal Bovine Serum (FBS; Merck), 1% PS, 0.1 mM 2-ME, 2 mM L-Gln, 2,000 U ml^−1^ Leukocyte Inhibitory Factor (LIF; Merck) and 1% (vol/vol) Nucleosides (Merck). The culture medium was replaced with fresh medium every day. To induce cortical neurons, the mESCs were replated in prime surface 96-well plates (Sumitomo Bakelite) at a density of 9,000 cells per well in differentiation medium containing GMEM (Thermo Fisher Scientific) supplemented with 10% (vol/vol) KnockOut Serum Replacement (KSR; Thermo Fisher Scientific), 0.1 mM MEM non-essential amino acids solution (NEAA; Thermo Fisher Scientific), 0.1 mM 2-ME, 1 mM sodium pyruvate solution (Pyruvate; Merck) and 2 mM L-Gln. 10 μM SB-431542 (Merck), which is a TGF-β receptor inhibitor, and 20 nM WNT-C59 (Collagen Technology, San Diego, CA, USA), which is a WNT inhibitor, were added by day 6. On day 7, we switched the medium from GMEM to DMEM/F12 (Fujifilm) supplemented with 0.1 mM 2-ME, 2 mM L-Gln, 1% N2 supplement and 2% B-27 supplement. Half of the medium was replaced with fresh medium every 3 days.

### Quantitative PCR (qPCR)

Total RNA was extracted from mouse embryos and cultured cells using the RNeasy Mini Kit (Qiagen, Valencia, CA, USA) or RNeasy Micro Kit (Qiagen). The cDNA was synthesized using the SuperScript III First-Strand Synthesis System (Thermo Fisher Scientific). The quantitative PCR (qPCR) was performed with SYBR Premix Ex Taq (Takara Bio) and with the Thermal Cycler Dice Real-Time System (Takara Bio). The data were analyzed using the delta-delta Ct method and normalized to *Gapdh* levels. Primers were designed by using prime3 plus, and the sequences were as follows: m*Gapdh*, forward 5′-TGTTCCTACCCCCAATGTGTC-3′, reverse 5′-TAGCCCAAGATGCCCTTCAG-3′; m*Foxg1*, forward 5′-ACCCTGCCCTGTGAGTCTTT-3′, reverse 5′-GACCCCTGATTTTGATGTGTG-3′; mR*eelin*, forward 5′-GCCACTGCTTACTCGCACCT-3′, reverse 5′-GCCACACTGCTCTCCCATCT-3′; m*Ctip2*, forward 5′-TTGGATGCCAGTGTGAGTTG-3′, reverse 5′-ATGTGTGTTCTGTGCGTGCT-3′; m*Cux1*, forward 5′-TCCTGGAACAAGCCAAGAGG-3′, reverse 5′-CTGTAGGATGGAGCGGATGG-3′; m*Nrp1*, forward 5′-CCGCCTGAACTACCCTGAAA-3′, reverse 5′-CACCCTGTGTCCCTACAGCA-3′; m*Tbr2*, forward 5′-TGTGACGGCCTACCAAAACA-3′, reverse 5′-GTACCGACCTCCAGGGACAA-3′; and m*Pax6*, forward 5′-GTGCCCTTCCATCTTTGCTT-3′, reverse 5′-CGCCCATCTGTTGCTTTTC-3′.

### Cell Sorting

Cell suspensions were prepared by using Accumax, and then the cells were resuspended in PBS containing 2% FBS, 20 mM D(+)-glucose (Fujifilm) and 1% PS. The samples were stained with anti-L1CAM antibody for 20 min. After primary antibody reactions, the samples were stained with Alexa fluorescent-conjugated antibodies (1:400) for 20 min. Dead cells were labeled with 7-amino-actinomycin D (7AAD; BD Biosciences). Cell sorting was performed using a FACS Aria II (BD Biosciences), and the data were analyzed with FACS Diva software (BD Biosciences).

After sorting, mouse cells were cultured in DMEM/F12 containing 2 mM L-Gln, 1% N2 supplement, 2% B-27 supplement, 1% PS, 20 ng ml^−1^ BDNF (Fujifilm), 10 μg ml^−1^ GDNF (Fujifilm) and 30 μM Y-27632. For *in vitro* studies, the sorted cells were cultured on chambered cell culture slides (Thermo Fisher Scientific) coated with poly-L-ornithine (50 μg ml^−1^, Merck), laminin (5 μg ml^−1^, Thermo Fisher Scientific) and fibronectin (5 μg ml^−1^, Merck). For *in vivo* studies, we cultured the sorted cells for 2 days before transplantation, because a lot of cells were dead or dying immediately after sorting and the efficiency was low and unstable. The sorted cells were replated in low cell adhesion 96-well plates at a density of 3 × 10^4^ cells per well. Half of the culture medium was replaced with fresh medium every 3 days.

### Microarray Analysis

Total RNA was extracted using the RNeasy Mini Kit. The samples were subjected to microarray analysis using GeneChip Mouse Gene 1.0 ST Arrays (Thermo Fisher Scientific). The arrays were scanned using the Microarray Scanner System (Agilent Technologies, Santa Clara, CA, USA). The data were analyzed using the GeneSpring software program (Agilent Technologies). The expression signals of the probe sets were calculated using RMA16. The microarray data are available from the Gene Expression Omnibus (GEO database) with the accession number GSE132362.

### EdU Incorporation Assay

Ten microgram EdU (Thermo Fisher Scientific) was added into the culture medium at 2 h before fixation. The detection of EdU incorporation into the DNA was performed with the Click-iT Plus Alexa Fluor 647 Cell Proliferation Assay Kit (Thermo Fisher Scientific). Fixed cells were incubated with 0.3% PBST for 30 min at RT. The Click-iT reaction cocktail was prepared according to the manufacturer’s instruction. The samples were incubated with the Click-iT reaction cocktail for 30 min at RT. After washing, the samples were subjected to immunostaining procedure.

### RNA Fluorescence *in situ* Hybridization (FISH)

Mouse embryos were fixed in PBS containing 4% PFA overnight at 4°C. Fixed samples were dehydrated in PBS containing 15% sucrose overnight at 4°C. Subsequently, the samples were sectioned with a cryostat at 16 μm thickness and attached to a MAS-coated slide glass. RNA FISH was performed using the RNAscope Multiplex Fluorescent v2 Kit (Advanced Cell Diagnostics Inc., Hayward, CA, USA). Sample slides were boiled with target retrieval buffer for 3 min, rinsed in 99.5% ethanol (Fujifilm) for 3 min, and then air-dried. The sample slides were subjected to protease digestion for 15 min at 40°C and incubated with RNAscope oligonucleotide probes (*Ctip2*, NM_021399.2) for 2 h at 40°C. After hybridization, the sample slides were incubated with AMP1 and AMP2 sequentially for 30 min each at 40°C. Subsequently, the sample slides were incubated with AMP3 for 15 min at 40°C. Finally, the sample slides were labeled with OPAL 590 (Perkin Elmer Japan Company Limited, Yokohama, Japan) for 30 min at 40°C. The reaction was stopped with an HRP blocker for 30 min at 40°C. After washing, the sample slides were subjected to immunostaining.

### Imaging and Data Analysis

Images were visualized using a fluorescence microscope (BZ-9000; Keyence, Osaka, Japan), In Cell Analyzer 6000 (GE Healthcare) and confocal laser microscope (Fluoview FV1000D; Olympus). To measure cell counts, immunopositive cells were manually counted for at least three independent samples to calculate the age of positive cells for each marker. The number of immunopositive cells in the graft was quantified in every 6 sections and corrected. To measure the graft size, low magnified GFP images were imported into the BZ-II Analyzer software (Keyence), and the graft areas were quantified every six sections. The estimated graft volumes were calculated based on the thickness of the brain slices. The number of axons derived from a graft was counted in the coronal section at the internal capsule and the cerebral peduncle. The site of interest in each animal was labeled using an anti-GFP antibody, and the mean number of axons was recorded.

### Statistical Analysis

Statistical analysis was performed using a software package (GraphPad Prism 7; GraphPad). Data from the *in vitro* and *in vivo* experiments were analyzed by Student’s *t*-test or one-way ANOVA with Bonferroni’s multiple comparison tests. The data were considered statistically significant for *p* < 0.05 and are shown as the mean ± standard error of the mean (SEM). All data were acquired from at least three independent experiments.

## Results

### The Frontal Cortex of E14.5 Mouse Contains CSMNs and Their Progenitors

**Figure 1:**
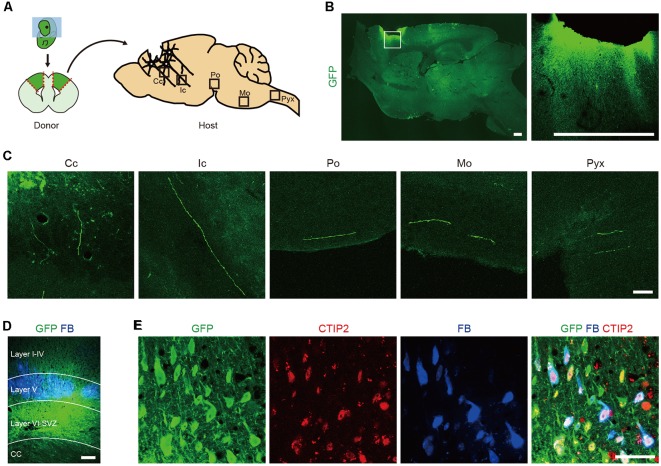
The frontal cortex of E14.5 mouse contains Corticospinal motor neurons (CSMNs) and their progenitors.** (A)** Schematic of the transplantation of fetal cortical tissue from E14.5 GFP Tg mice into the lesion cavity of adult mice. **(B)** After 2 months, the transplanted cells survive and extend axons into the host brain, as shown by GFP^+^ fiber innervations. Scale bars represent 500 μm. **(C)** GFP^+^ fibers were found in the corpus callosum (Cc), internal capsule (Ic), pons (Po), medulla oblongata (Mo) and pyramidal decussation (Pyx). Scale bar represents 50 μm. **(D)** FB was injected into the pyramidal decussation at 7 days before sacrifice. GFP^+^/FB^+^ cells were found in the cortical layer V. Scale bar represents 200 μm. **(E)** Immunofluorescence images of a graft stained with anti-GFP (green) and anti-CTIP2 (red) antibodies. GFP^+^/FB^+^ cells expressed CTIP2. Scale bar represents 50 μm.

To identify which cells extend axons along the CST, we isolated the cerebral cortices of GFP transgenic (Tg) mice at embryonic day (E) 14.5 (Okabe et al., 1997) and transplanted the dissociated tissue into the frontal lobe of adult mice (Figure 1A). Two months after the transplantation, we performed immunohistological analyses of the brain. GFP^+^ graft-derived fibers were observed along the CST at the corpus callosum, internal capsule, pons, medulla oblongata and pyramidal decussation (Figures 1B,C). Seven days prior to sacrifice, we injected a retrograde axonal tracer, FB, into the pyramidal decussation and found it labeled cells in layer V of the frontal lobe (Figure 1D). This observation is consistent with CSMNs residing in cortical layer V. A subpopulation of FB^+^ cells expressed GFP, and all GFP^+^/FB^+^ cells expressed CTIP2, which is a marker for layer V neurons and plays a critical role in the development of CSMN axonal projections to the spinal cord (Arlotta et al., 2005; Figure 1E). These results indicate that the frontal cortex of E14.5 mouse contains cells that extend their axons along the CST and that these cells express CTIP2.

### Mouse ESC-Derived CTIP2:GFP^+^ Cells Have Characteristics of CSMNs

**Figure 2:**
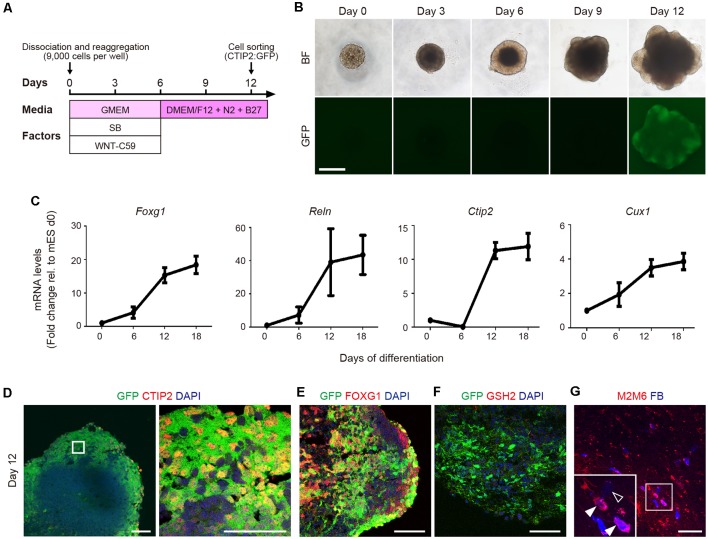
Mouse ESC-derived CTIP2:GFP^+^ cells show characteristics of CSMNs.** (A)** Schematic diagram of the cortical differentiation protocol from mouse embryonic stem cells (mESCs). **(B)** Bright-field and CTIP2:GFP images of the floating culture of cell aggregates from day 0 to day 12. The GFP signal is detected from day 12 onwards. Scale bar represents 500 μm. **(C)** Gene expression analysis for Foxg1, Reln, Ctip2 and Cux1 by qRT-PCR (*n* = 4). All values are displayed as means ± SEM. **(D–F)** Immunofluorescence images of CTIP2:GFP KI mESC aggregates for GFP (green), CTIP2 (red), FOXG1 (red), GSH2 (red) and DAPI (blue) on day 12. Scale bars represent 50 μm.** (G)** Immunofluorescence image of a graft stained with anti-M2/M6 (red) antibodies. FB (blue) shows host cells (M2M6^−^/FB^+^, open arrowhead) and graft-derived cells (M2M6^+^/FB^+^, filled arrowhead). Scale bar represents 100 μm.

To investigate the characteristics of CTIP2^+^ cells, we generated CTIP2:GFP knock-in (KI) mESCs and differentiated them into neural lineage by inhibiting WNT and TGF/Activin/Nodal signaling in a floating culture of cell aggregates (Figure 2A; Motono et al., 2016). The sphere size gradually increased, and GFP expression became detectable by around day 12 (Figure 2B). Temporal gene expression analyses revealed that the mRNA levels of *Foxg1* (telencephalic progenitors), *Reelin* (layer I), *Ctip2* (deep layer) and *Cux1* (upper layer) were gradually increased along with the differentiation (Figure 2C). Immunofluorescence studies revealed that >95% of the GFP expression of day-12 spheres was colocalized with the expressions of CTIP2 and FOXG1 (Figures 2D,E). In the telencephalon of the mouse embryo, CTIP2 is expressed not only in the cerebral cortex but also in the basal ganglia (Leid et al., 2004). However, we did not find cells that expressed GSH, a marker for the lateral ganglionic eminence (Figure 2F), thus confirming CTIP2^+^ cells are cortical cells.

To determine whether mESC-derived CTIP2:GFP^+^ cells have the characteristics of CSMNs, we isolated CTIP2:GFP^+^ cells by fluorescence-activated cell sorting (FACS) on day 12. After incubation for 2 days, we injected the CTIP2:GFP^+^ cell aggregates into the frontal lobe of adult nude rats. Three months after transplantation, we injected FB into the pyramidal decussation, and 7 days later, the rats were subjected to an immunohistological study. Grafted cells were identified by the expression of M2/M6, a specific marker for mouse cell membrane (Lagenaur and Schachner, 1981; Lagenaur et al., 1992), and the FB signal was observed in the M2^+^/M6^+^ cells, suggesting that the sorted cells show characteristics of CSMNs (Figure 2G).

### mESC-Derived CTIP2:GFP^+^ Cells Express L1CAM

**Figure 3:**
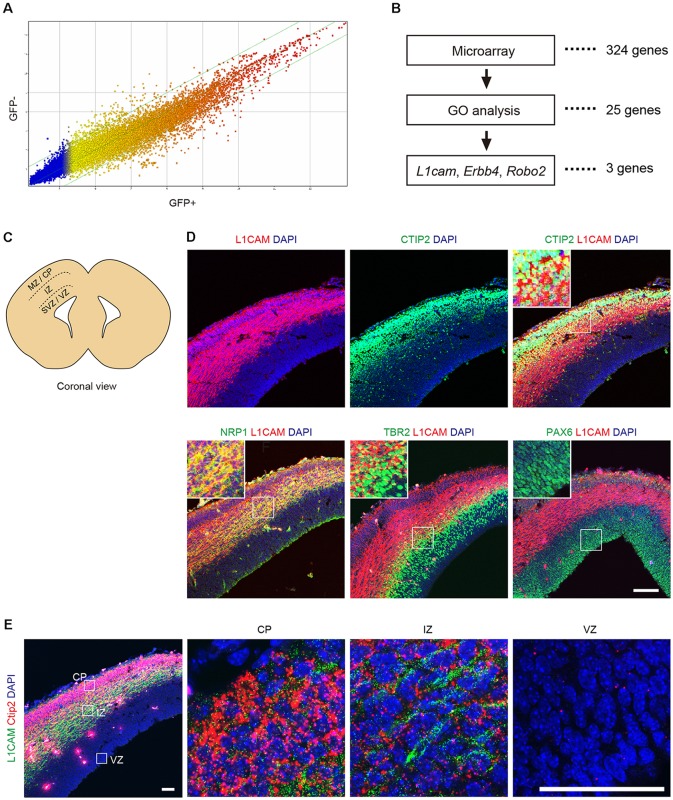
L1CAM is a marker for CTIP2 of E14.5 mouse.** (A)** Comparison of the gene expression profiles between GFP^+^ and GFP^−^ cells in CTIP2:GFP KI mESCs on day 11 to day 13. **(B)** Screening of the candidate molecules by microarray analysis, gene ontology (GO) analysis and literature. First, the microarray analysis revealed 324 genes as 2-fold upregulated in CTIP2:GFP^+^ cells. Next, 25 genes were identified by GO analysis as the plasma membrane. Finally, three genes were selected from the literature as expressed in the cell body. **(C)** Diagram of the brain slices of the E14.5 mouse frontal cortex. **(D)** Immunofluorescence images for CTIP2 (green), NRP1 (green), TBR2 (green), PAX6 (green), L1CAM (red) and DAPI (blue). Scale bar represents 100 μm. **(E)** Immunofluorescence image for L1CAM (green) and RNA FISH image for *Ctip2* (red) and DAPI (blue). Scale bars represent 50 μm.

To identify a cell surface marker for CTIP2:GFP^+^ cells, the differentiated cells were divided into GFP^+^ and GFP^−^ cells by FACS on day 11 to day 13 (see Supplementary Figure S1) and were subjected to microarray analysis. A gene expression profile revealed that 324 genes were up-regulated more than 2-fold in the CTIP2:GFP^+^ population (Figure 3A). Classification of the up-regulated genes by a Gene Ontology (GO) analysis showed that 25 genes encoded plasma membrane-related proteins. Among them, we chose six candidates (Erbb4, Grin2b, Robo2, Erc2, Dlg2, L1cam) for which antibodies are commercially available. Then, we excluded three candidates (Grin2b, Erc2, Dlg2) because they are mainly expressed in synapses and axons (Moriyoshi et al., 1991; Ohtsuka et al., 2002; Tao et al., 2003). We finally selected three genes that code proteins expressed on the surface of the cell body: Roundabout guidance receptor 2 (*Robo2*), Erb-b2 receptor tyrosine kinase 4 (*Erbb4*) and L1 cell adhesion molecule (*L1cam*; Figure 3B).

Robo2 is a member of the immunoglobulin superfamily of cell adhesion molecules and acts as a guidance receptor by binding secreted SLIT ligands (Holmes et al., 1998; Long et al., 2004). In the developing cerebral cortex, Robo2 is distinctly expressed in the intermediate zone (IZ), where prospective interneurons and projection neurons migrate tangentially from the ventricular zone (VZ) and the subventricular zone (SVZ) to the cortical plate (CP; Andrews et al., 2007; López-Bendito et al., 2007). Erbb4 is a member of the type I receptor tyrosine kinase subfamily and is involved in cell proliferation, migration and differentiation (Burden and Yarden, 1997; Adlkofer and Lai, 2000; Buonanno and Fischbach, 2001). In the developing cerebral cortex, Erbb4 is expressed in the IZ (Yau et al., 2003). The protein is preferentially expressed in parvalbumin^+^ interneurons and subsets of other GABAergic interneurons in the adult cerebral cortex. L1CAM is a transmembrane glycoprotein composed of six immunoglobulin domains and five fibronectin type III repeats (Schachner, 1991). In mammals, L1CAM is expressed throughout the nervous system and is involved in axon growth and guidance during development, interactions between Schwann cells and axons, neuronal cell migration, neuronal survival, synaptogenesis and myelination (Lindner et al., 1983; Bixby et al., 1988; Chen et al., 1999). In the adult cerebral cortex, L1CAM is specifically localized in cortical layer V (Munakata et al., 2003). Based on these characteristic features, we decided to focus on L1CAM as a candidate surface marker of CTIP2:GFP^+^ cells.

### Sorting of L1CAM^+^ Cells Enriches Cortical and Migrating Neurons

Next, we examined the expression pattern of L1CAM in the brain of E14.5 mice (Figure 3C). An immunofluorescence study revealed that L1CAM was expressed in the CP (CTIP2^+^), IZ (NRP1^+^), and upper part of the SVZ (TBR2^+^), but not in the VZ (PAX6^+^; Figure 3D). Moreover, we found that CTIP2 is expressed not only in the CP but also in the IZ. To confirm this observation, we performed fluorescence *in situ* hybridization (FISH) using the *Ctip2* mRNA probe combined with immunostaining for L1CAM. As expected, an intense Ctip2 signal was observed in the CP, and a weaker but clear signal was observed in the IZ (Figure 3E).

**Figure 4:**
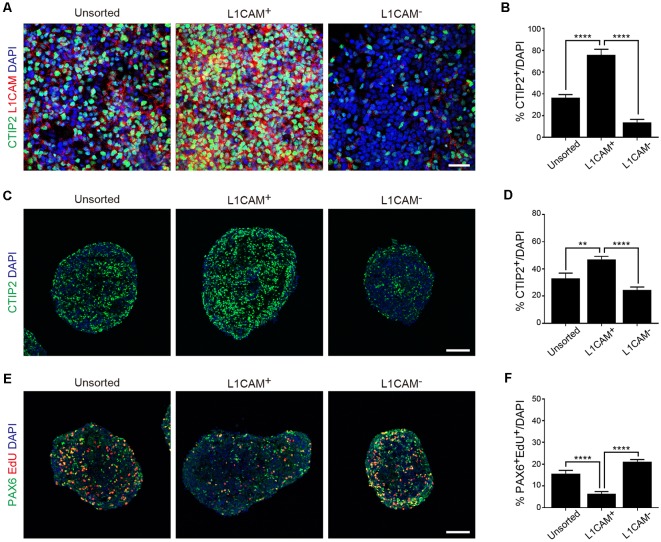
Sorting of L1CAM^+^ cells enriches cortical and migrating neurons. The frontal cortex was taken from E14.5 mice and used for *in vitro* studies. **(A)** Immunofluorescence images of the cells from unsorted, L1CAM^+^ and L1CAM^−^ cells for CTIP2 (green), L1CAM (red) and DAPI (blue) several hours after sorting. Scale bar represents 30 μm. **(B)** Percentage of CTIP2^+^ cells in total cells stained with DAPI (unsorted: *n* = 6; L1CAM^+^: *n* = 6; L1CAM^−^: *n* = 6). **(C)** Immunofluorescence images of unsorted, L1CAM^+^ and L1CAM^−^ cells for CTIP2 (green) and DAPI (blue) 2 days after sorting. Scale bar represents 100 μm. **(D)** Percentage of CTIP2^+^ cells in total cells stained with DAPI (unsorted: *n* = 9; L1CAM^+^: *n* = 9; L1CAM^−^: *n* = 8). **(E)** Immunofluorescence images of unsorted, L1CAM^+^ and L1CAM^−^ cells for PAX6 (green), EdU (red) and DAPI (blue) 2 days after sorting. 10 μM EdU was added in the culture medium 2 h before fixation. Scale bar represents 100 μm. **(F)** Percentage of PAX6^+^/EdU^+^ cells in total cells stained with DAPI (unsorted: *n* = 9; L1CAM^+^: *n* = 9; L1CAM^−^: *n* = 8). All values are displayed as means ± SEM. One-way ANOVA with Bonferroni’s multiple comparison test, **P < 0.01 and ****P < 0.0001.

Next, we dissociated the cortical tissue of E14.5 mice and divided it into L1CAM^+^ and L1CAM^−^ cells by FACS. An immunofluorescent study revealed that CTIP2^+^ cells were more frequently observed in the L1CAM^+^ population than the unsorted or L1CAM^−^ population (75.8 ± 5.0% vs. 36.4 ± 2.9% or 13.9 ± 2.7%, respectively; *n* = 6; Figures 4A,B). A quantitative reverse transcriptase-polymerase chain reaction (qPCR) analysis revealed that L1CAM^+^ cells expressed higher levels of Ctip2 and Nrp1 mRNAs compared to L1CAM^−^ cells (see Supplementary Figure S2). On the other hand, the mRNA expression levels of Tbr2 and Pax6 were lower in L1CAM^+^ cells (see Supplementary Figure S2). When we continued the culture as spheres for 2 days after sorting, CTIP2^+^ cells were again more frequently observed in the L1CAM^+^ population than the unsorted or L1CAM^−^ populations (47.2 ± 2.0% vs. 32.9 ± 4.0% or 24.5 ± 2.1%, *n* = 9, 9 and 8, respectively; Figures 4C,D). On the other hand, the L1CAM^+^ population contained fewer EdU^+^/PAX6^+^ proliferating cells compared to the unsorted or L1CAM^−^ populations (6.4 ± 1.0% vs. 15.6 ± 1.6% or 21.1 ± 1.0%, *n* = 9, 9 and 8, respectively; Figures 4E,F). These results suggested that sorting for L1CAM+ cells can be a strategy for efficient and safe cell therapy by enriching CSMNs and eliminating proliferating progenitor cells.

### L1CAM^+^ Cells Preferentially Extend Axons Along the CST

To investigate the survival and neurite extension of transplanted L1CAM^+^ cells *in vivo*, we dissected cortical tissues from E14.5 GFP Tg mice and divided them into L1CAM^+^ and L1CAM^−^ cells by using FACS. At 2 days after sorting, we transplanted the cell aggregates into the frontal cortex of adult mice. FB was injected into the pyramidal decussation at 2 months after transplantation, and 7 days later, these mice were subjected to immunohistochemical analyses.

**Figure 5:**
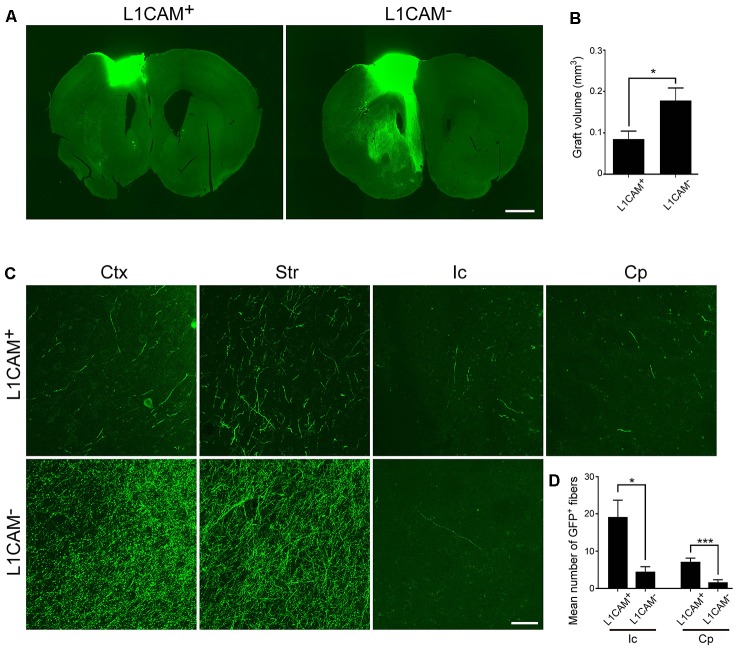
L1CAM^+^ cells survive and extend axons along the corticospinal tract (CST). L1CAM^+^ cells and L1CAM^−^ cells were isolated from E14.5 mouse frontal cortex, and 2 days later they were injected into the adult brain for 2 months. **(A)** Immunofluorescence images of the graft for GFP (green). Scale bar represents 1 mm. **(B)** Quantification of the volume of L1CAM^+^ cells and L1CAM^−^ cells in the grafts (L1CAM^+^: *n* = 6 and L1CAM^−^: *n* = 6).** (C)** Immunofluorescence images of the graft fibers for GFP (green). GFP^+^ fibers were found in the cerebral cortex (Ctx), striatum (Str), internal capsule (Ic) and cerebral peduncle (Cp). Scale bar represents 50 μm. **(D)** Quantification of GFP^+^ fibers from L1CAM^+^ cells and L1CAM^−^ cells (L1CAM+: *n* = 6 and L1CAM−: *n* = 6) at the Ic and Cp. All values are displayed as means ± SEM. Student’s *t*-test, *P < 0.05 and ***P < 0.001.

**Figure 6:**
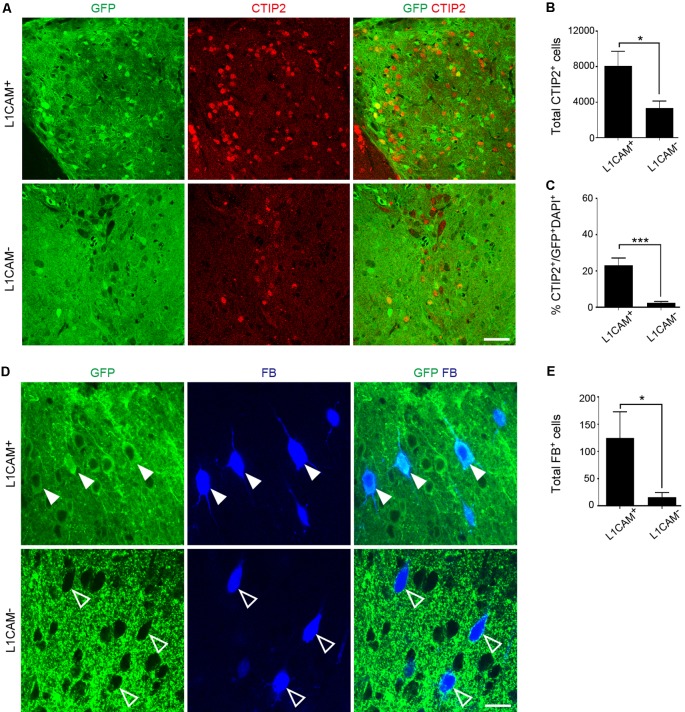
L1CAM^+^ grafts have a character consistent with CSMNs. L1CAM^+^ cells and L1CAM^−^ cells were isolated from E14.5 mouse frontal cortex, and 2 days later they were injected into the adult brain for 2 months. **(A)** Immunofluorescence images of the graft for GFP (green) and CTIP2 (red). Scale bar represents 50 μm. **(B)** Total number of CTIP2^+^ cells in the L1CAM^+^ grafts and L1CAM^−^ grafts (L1CAM^+^: *n* = 6 and L1CAM^−^: *n* = 6). **(C)** Percentage of CTIP2^+^ cells in total cells stained with DAPI and GFP (L1CAM^+^: *n* = 6 and L1CAM^−^: *n* = 6).** (D)** Immunofluorescence images of graft for GFP (green) and FB (blue). FB was injected into the pyramidal decussation 7 days before sacrifice. Solid white arrowheads represent graft-derived cells. Open arrowheads represent host-derived cells. Scale bar represents 25 μm. **(E)** Total number of FB^+^ cells in L1CAM^+^ grafts and L1CAM^−^ grafts (L1CAM^+^: *n* = 6 and L1CAM^−^: *n* = 6). All values are displayed as means ± SEM. Student’s *t*-test, *P < 0.05 and ***P < 0.001.

Immunostaining for GFP revealed that the size of the L1CAM^+^ grafts was significantly smaller than that of the L1CAM^−^ grafts (0.09 ± 0.02 mm^3^ vs. 0.18 ± 0.03 mm^3^, respectively; *n* = 6; Figures 5A,B). The axons from the L1CAM^+^ grafts were observed along the CST including the internal capsule and cerebral peduncle (Figures 5C,D). On the other hand, those from the L1CAM^−^ grafts were observed in the ipsilateral cortex or restricted within the striatum. The total number and percentage of CTIP2^+^ cells were higher in the L1CAM^+^ grafts than in the L1CAM^−^ grafts (Figures 6A–C). Furthermore, FB^+^ cells were more frequently observed in L1CAM^+^ grafts than L1CAM^−^ grafts (125 ± 48 cells vs. 16 ± 8 cells, respectively; *n* = 6; Figures 6D,E). On the other hand, CUX1^+^ cells (upper neurons) were more frequently observed in L1CAM^−^ grafts (see Supplementary Figure S3), but the cell density was not significantly different between the two grafts (see Supplementary Figure S3). These results indicate that L1CAM^+^ grafts contained more CSMNs and preferentially extended axons along the CST.

## Discussion

In this study, we grafted fetal brain tissue into the mouse brain and found that the cells in the graft extending axons along the CST expressed CTIP2. By using CTIP2:GFP KI mESCs, we identified L1CAM as a cell surface marker for cortical CTIP2^+^ cells. Finally, we sorted L1CAM^+^ cells of the fetal brain and confirmed that these cells more efficiently extended axons along the CST compared to L1CAM^−^ cells.

As mentioned above, L1CAM is a transmembrane glycoprotein composed of six immunoglobulin domains and five fibronectin type III repeats (Schachner, 1991). In mammals, L1CAM is expressed throughout the nervous system and is involved in axonal growth and guidance during development (Lindner et al., 1983; Bixby et al., 1988; Chen et al., 1999).

In a developing mouse brain, cortical neurons are differentiated from progenitor cells that line the dorsal aspect of the lateral ventricles in the forebrain (Nieto et al., 2004; Leone et al., 2008). Proliferating progenitor cells that express PAX6 are found in the VZ, which is immediately adjacent to the ventricles, and at later stages in the SVZ, which forms between the VZ and the overlying IZ. The immature neurons exit the cell cycle at around E10, migrate out of the VZ along radial glia and reach the CP. Within the CP, neurons of the deep layers (VI and V) are generated at around E14, and neurons of the upper layers (IV, III and II) are generated at around E16. The deep layers are mainly composed of subcerebral projection neurons, which express CTIP2 and extend axons beyond the cortex such as the thalamus and spinal cord. In contrast, the upper layers are mainly composed of callosal projection neurons, which express SATB2 and CUX1 and extend axons to the other cortical area.

This complexity of the developing brain has made it difficult to identify which cell populations contribute to the reconstruction of the CST after transplantation. A previous transplantation study of E15.5 mouse brain revealed that early postmitotic neurons, which are fate-restricted for deep-layer neurons, can extend axons as CST and establish functional connectivity after transplantation (Wuttke et al., 2018). These neurons underwent final mitosis during E11.5-13.5 and were postmitotic at the time of transplantation. In contrast, the cells dividing at E14.5 did not differentiate into CTIP2^+^ cells in the deep layers. In the present study, we found that L1CAM^+^ cells of E14.5 mouse brain more efficiently extended axons along the CST compared to L1CAM^−^ cells. Intriguingly, the former cell population contained more postmitotic CTIP2^+^ cells, while the latter contained more proliferating PAX6^+^ progenitor cells. This distinction suggests sorting for L1CAM^+^ cells of the E14.5 mouse brain could enrich cells that contribute to the reconstruction of the CST. In this context, the timing of sorting during neuronal development is critical. To enrich CSMNs, L1CAM^+^ cells need to be sorted at the early cortical development when only deep layer neurons emerged.

We previously reported that neuropilin-1 (NRP1)^+^ cells in the frontal cortex of E14.5 mice survive and extend axons to the spinal cord of neonatal brain (Sano et al., 2017). NRP1 is a Sema 3 receptor, is essential for the initial stage of axonal sprouting (Bagnard et al., 1998; Fujisawa, 2004), and is distributed in the IZ of the cerebral cortex of E13.5-15.5 mouse (Kawakami et al., 1996; Hatanaka et al., 2009). NRP1^+^ cells are mainly migrating neurons in the IZ, but they are also subcortical projection neurons with axonal extensions. In this study, we found L1CAM by microarray analysis using CTIP2:GFP KI mESCs as a cell surface marker to enrich cells that extend axons along the CST. In addition, L1CAM and NRP1 form a complex as a Sema 3A receptor for transducing signaling pathways within the growth cone (Castellani et al., 2000). These facts suggest that L1CAM^+^/NRP1^+^ cells can efficiently contribute to the reconstruction of the CST, but it remains unknown whether deep layer neurons in the CP or migrating neurons in the IZ play a more important role.

Another advantage of sorting L1CAM^+^ cells is that one can eliminate proliferating progenitor cells that are unlikely to become subcerebral projection neurons or have tumorigenicity. Tumorigenicity is especially a concern in the case of transplantation using PSC-derived cells.

In contrast to L1CAM^+^ grafts, L1CAM^−^ grafts extended axonal fibers mainly to the ipsilateral cortex and striatum. Additionally, only a few fibers were observed in other areas including the thalamus and superior colliculus in both cases. For a complete analysis of fiber extension in the brain, we need to employ the brain tissue-clearing and 3D-imaging technique, which is the next challenge in the near future.

In conclusion, we demonstrated that sorting L1CAM^+^ cells from the cerebral cortex of E14.5 mouse is advantageous for enriching cells that can extend axons along the CST after transplantation. However, it remains to be explored whether these cells have a functional effect on mouse behavior after transplantation. In addition, it is unknown whether the same strategy can be applied to human PSC-derived neurons. Recently, we have published a article about human PSC-derived cerebral organoids (Sakaguchi et al., 2019). For clinical application, we need to determine the optimal timing for sorting in the induction of human cerebral organoids and examine if human L1CAM^+^ cells contribute to safe and efficient transplantation. An investigation into these questions will advance cell-based therapies to treat CST damaged by stroke or brain injury.

## Data Availability Statement

The datasets generated for this study can be found in the microarray data available from the Gene Expression Omnibus (GEO database) with the accession number GSE132362.

## Ethics Statement

The animal study was reviewed and approved by The animal experimentation committee of Center for iPS Cell Research and Application (CiRA), Kyoto University.

## Author Contributions

BS and JT designed the study and wrote the manuscript. BS, RT, YI, and KF performed the experiments and analyzed the data. HN and YO generated the transgenic mESC clones.

## Conflict of Interest

The authors declare that the research was conducted in the absence of any commercial or financial relationships that could be construed as a potential conflict of interest.
