# Supplementary material for: L1CAM Is a Marker for Enriching Corticospinal Motor Neurons in the Developing Brain
Source: Front Cell Neurosci. 2020 Feb 19;14:31. doi: 10.3389/fncel.2020.00031 (PMC7042175; doi:10.3389/fncel.2020.00031)
Supplement: Supplementary file 1 [file Data_Sheet_1.pdf]

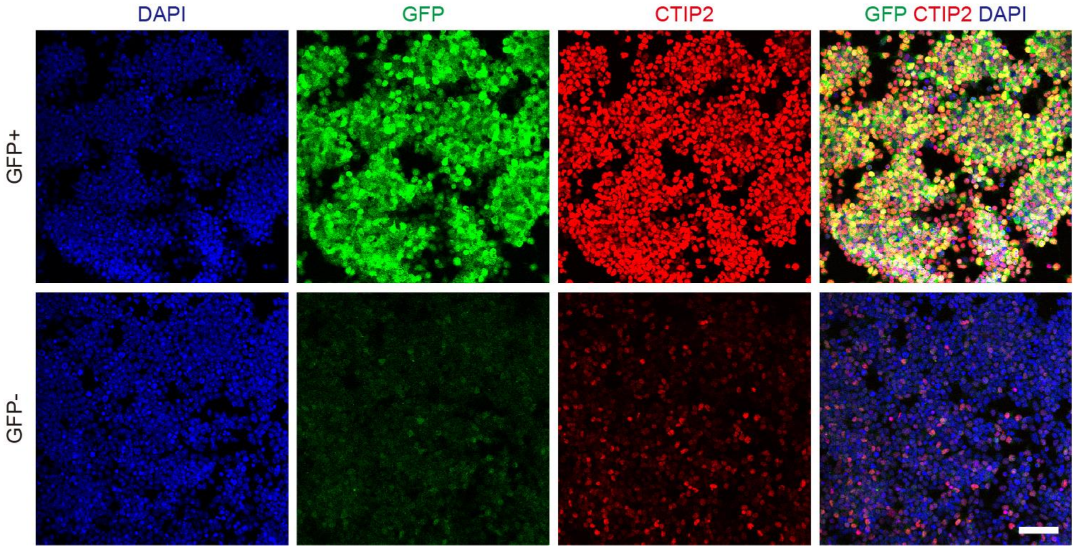

Figure S1. Purification of $CTIP2^+$ cells by the expression of CTIP2:GFP. Immunofluorescence images for GFP (green), CTIP2 (red) and DAPI (blue) immediately after sorting. $CTIP2^+$ cells were enriched in the $CTIP2:GFP^+$ fraction. Scale bar represents 50 μm.

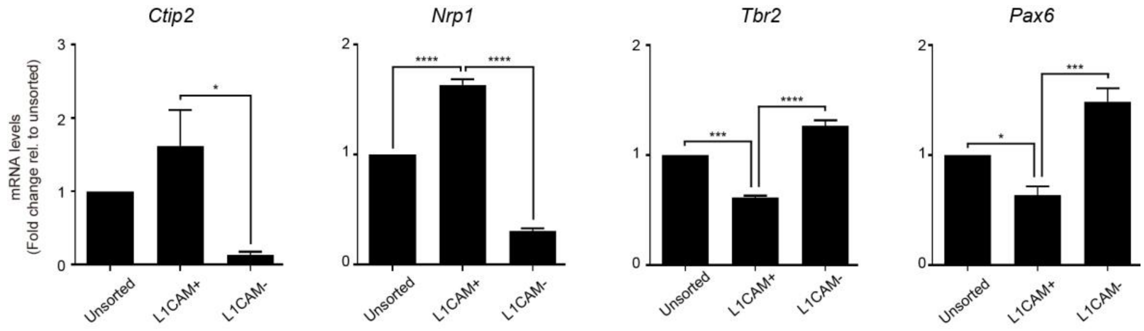

Figure S2. Gene expression analysis of FACS-purified cells. E14.5 mouse frontal cortex was separated into unsorted, $L1CAM^+$ and $L1CAM^-$ fractions (unsorted: $n = 3$; $L1CAM^+$: $n = 3$; $L1CAM^-$: $n = 3$). Gene expression analysis was performed by qRT-PCR with *Ctip2*, *Nrp1*, *Tbr2* and *Pax6* primers. All values are displayed as means ± s.e.m. One-way

ANOVA with Bonferroni's multiple comparison tests, *P<0.05, ***P<0.001 and ****P<0.0001.

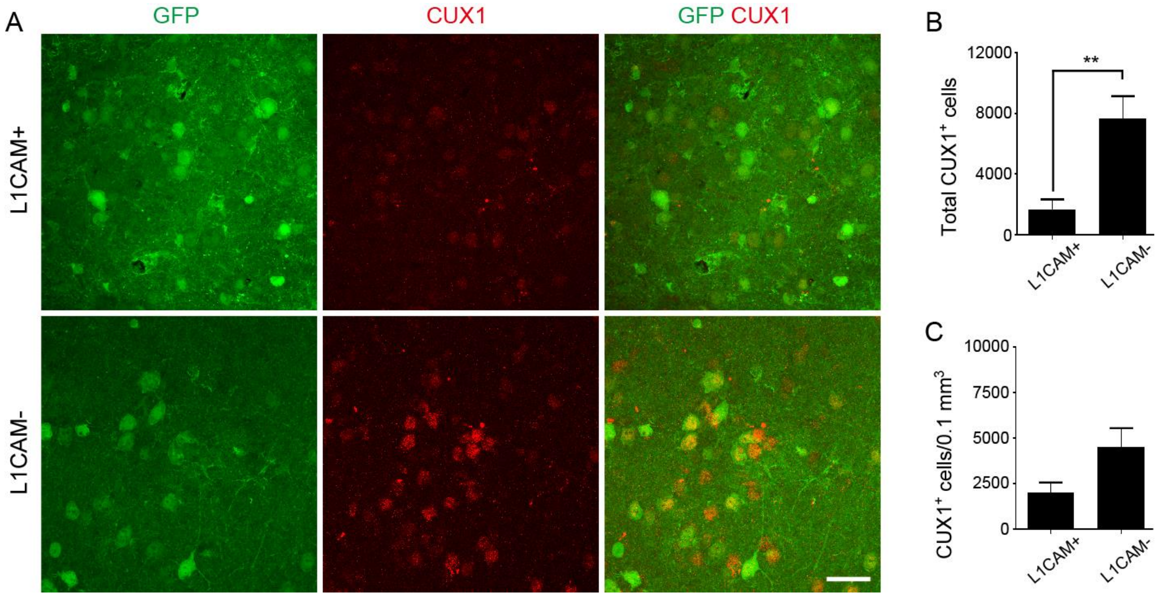

Figure S3. L1CAM$^+$ cells and L1CAM$^-$ cells were isolated from E14.5 mouse frontal cortex, and two days later they were injected into adult brain for two months. **(A)** Immunofluorescence images of the graft for GFP (green) and CUX1 (red). Scale bar represents 50 μm. **(B)** Total number of CUX1$^+$ cells in L1CAM$^+$ grafts and L1CAM$^-$ grafts (L1CAM$^+$: n = 6 and L1CAM$^-$: n = 6). **(C)** CUX1$^+$ cell densities in L1CAM$^+$ grafts and L1CAM$^-$ grafts (L1CAM$^+$: n = 6 and L1CAM$^-$: n = 6). All values are displayed as means ± s.e.m. Student's t-test, **P<0.01.
